# Investigations on the Performances of Corn Starch/PBAT Blends

**DOI:** 10.3390/polym18060767

**Published:** 2026-03-21

**Authors:** Wenzhuo Zhao, Rui Qiu, Miaoyi Fang, Wen Lei, Yong Chen

**Affiliations:** 1 College of Science, Nanjing Forestry University, Nanjing 210037, China; 2 Shanghai Hongrui Biotechnology Co., Ltd., Shanghai 201199, China

**Keywords:** poly (butylene adipate-co-terephthalate), corn starch, blend, degradation, cost

## Abstract

Corn starch (CS)/poly (butylene adipate-co-terephthalate) (PBAT) blends were prepared by extrusion and injection molding processes. The CS content in the blends changed between 0 and 50 wt.% in 10 wt.% steps. Melt flow rates, mechanical properties, thermal stability, melting and crystallization behavior, as well as hydrophilicity of the blends were investigated. Based on these, the degradation properties of PBAT and the blend containing 50 wt.% CS (50%CS/PBAT) in water and open-air storage were comparatively studied via visual appearance observation, Shore hardness testing, and water absorption measurement. The results showed that the melt flow rates and the mechanical properties of the blends, including the tensile strength, tensile modulus, impact strength, and elongation at break, initially increased before decreasing as CS content in the blends increased, while the flexural strength and flexural modulus of the samples increased monotonously. The sample would become more thermal unstable when more CS was used. Besides these, the crystallinity and water contact angle became smaller. Immersion in water would blacken the visual appearances of PBAT and 50%CS/PBAT samples, but cracks could be found much more obviously in the blend than in neat PBAT; both the hardness and the mass of PBAT rose slightly while those of 50%CS/PBAT dropped significantly. An open-air storage would also blacken the visual appearances of PBAT and 50%CS/PBAT, and the hardness of the two samples would be decreased to almost the same extent. The results showed that the incorporation of CS in PBAT had much greater effects on the flow ability, mechanical properties, thermal stability, melt and crystallization behavior, as well as hydrophilicity of the blends. Immersion in water or being placed in air could accelerate the degradation of 50%CS/PBAT much more seriously than PBAT. Compared with PBAT, 50%CS/PBAT was of much lower cost and easier to be degraded, especially in water; it should be an ideal degradable blend for applications in packaging, agricultural mulch, and some other areas.

## 1. Introduction

As a typical biodegradable block-copolyester, poly (butylene adipate-co-terephthalate) (PBAT) can be completely degraded into the end products of carbon dioxide and water. This copolymer is always prepared by chemical synthesis from fossil resources [1]. Its inherent advantages, especially the remarkable flexibility and excellent toughness [2,3], makes it become an ideal candidate in the fields of agriculture, packaging films, and medical devices. However, the production method, high production cost, weak gas barrier properties, and relatively low modulus and stiffness of PBAT have hindered its progress toward a bright future of sustainable production and growth [4,5]. In addition, it generally degrades very slowly under natural conditions for over two years [6]. It is quite necessary to make some improvements in copolymer’s properties and lower the material costs. Blending with starch should stand out as a highly promising option for this purpose.

Starch is a highly economical raw material and admirable oxygen resistant [7]; it can be processed as an affordable substrate for innovative biodegradable polymers. Numerous endeavors have been undertaken to blend starch with some degradable polymers. For example, Ordoñez et al. [8] designed three-layered films—PLA/starch/PLA—for active packaging. The food shelf life could be extended using these films. Kurup et al. [9] investigated the optimized injection molding parameters for a novel tapioca starch/PLA blend using response surface methodology. The elongation, flexural strength, and impact strength were comparable to conventional plastics. The enhanced biodegradation test under ambient soil conditions showed no leaching in most stimulants, supporting its potential for sustainable packaging applications. Liu et al. [10] compared the different properties of polybutylene succinate (PBS) blending with three types of esterified starch with different amylose content, indicating the esterified waxy corn starch/PBS composite. The one with the lowest amylose content had the best hydrophobicity and degradation performance. Corrêa et al. [11] plasticized starch with urea and then blended it with poly (ε-caprolactone) (PCL), using PCL grafted with maleic anhydride (PCL-g-MA) as a compatibilizing agent. The biodegradability of the films prepared by the blend was investigated by characterizing its physico-mechanical properties. They found that the films were flexible and presented biodegradability and nitrogen release as a function of starch content on blends formulation. The blends could be processed into mulching films in agricultural area. Eslami et al. [12] developed a thin trilayer structure assembly of two outer layers of poly(3-hydroxybutyrate-co-3-hydroxyvalerate) (PHBV), as well as a core layer of maleated starch/PHBV blend (80/20). Its modulus was as high as 178 MPa, while the water vapor transmission rate was only 20.2 g/(m^2^·d). Meanwhile, the oxygen permeation could not be detected. This biodegradable trilayer structure with excellent barrier properties was supposed to be an excellent food packaging material.

Up to now, however, few works have been done on the degradation behaviors of starch reinforced bioplastic in water or open-air storage. To compensate for this deficiency, in this paper, we first prepared starch/PBAT blends using both extrusion and the injection molding process, and we investigated the performances of the blends containing different amount of starch. Then, the degradation behaviors of the blends containing 50 wt.% starch in water and open-air storage were investigated individually; at the same time, neat PBAT was used for comparison. The ultimate aim of this study is to reduce the cost of PBAT, improve its comprehensive properties, and promote its application in more areas.

## 2. Experimental

### 2.1. Materials and Reagents

**Figure 1:**
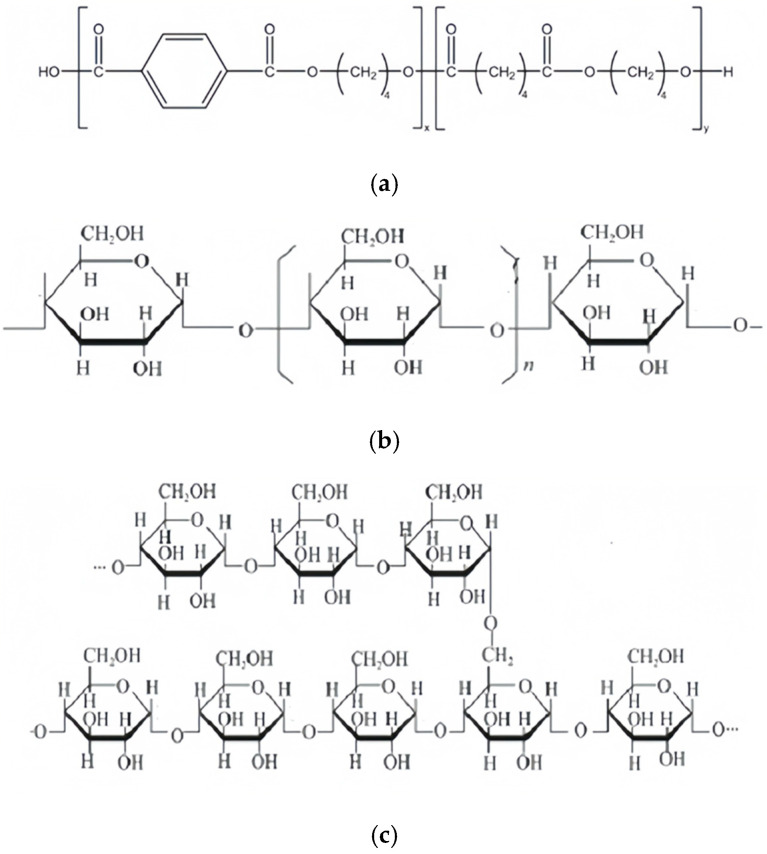
Chemical structures of PBAT and CS, (**a**) PBAT, (**b**) amylose, (**c**) amylopectin.

PBAT in pellet form was purchased from Xinjiang Blue Ridge Tunhe Sci. & Tech. Co., Ltd., China (Changji, China); its chemical structure is illustrated in Figure 1a. Corn starch (CS), 80 mesh, was purchased from Shandong Hengreng Industrial and Commercial Trading Co. Ltd. China (Tengzhou, China). CS was composed of amylose and amylopectin, whose chemical structures are shown in Figure 1b and Figure 1c, respectively.

### 2.2. Sample Preparation

The dried PBAT and starch were poured into a container and well-mixed at room temperature. Then, the mixture was fed into the extruder supplied by Nanjing Hongjiayuan Machinery Co. Ltd. (SHJ-25, Nanjing, China) using a gravimetric feeder, extruded and pelletized with a 100 rpm screw speed and a barrel temperature of 105–120 °C. Subsequently, the granulated CS/PBAT were injection molded using an electric injection molding machine (CWI-90BV, Shanghai Jiwei Machinery Industry Co. Ltd., Shanghai, China) to obtain the samples for testing. The injection temperature was controlled in the range from 120 °C to 150 °C, and the injection pressure was kept at 140 MPa.

The samples containing 10 wt.% CS, 20 wt.% CS, 30 wt.% CS, 40 wt.% CS, and 50 wt.% CS were defined as 10%CS/PBAT, 20%CS/PBAT, 30%CS/PBAT, 40%CS/PBAT, and 50%CS/PBAT, respectively.

### 2.3. Degradation Tests

The samples of 50%CS/PBAT and neat PBAT were both divided into two groups. All the samples in one group were immersed in water held in a open plastic container, and then they were moved ourdoors; all the samples in the other group were placed on outdoor concrete ground in air directly. The degradation tests of the samples in water or an open-air storage were conducted from 1 July to 31 August 2025 in Nanjing, Jiangsu, China, lasting for 60 days. It was sunny for approximately 16 days, cloudy for 32 days, and rainny for 12 days. The temperature ranged from 24 °C to 37 °C, as reported by the local meteorological department. Changes in appearance, weight, and hardness were investigated.

### 2.4. Testing and Characterization

#### 2.4.1. Melt Flow Rate Testing

The melt flow rate (MFR) was measured according to the reference Chinese national standard GB/T 3682.1-2018 [13] under the conditions of 190 °C and 2.16 kg, using a melt flow rate tester (ZRZ1452, Meister Industrial Systems, Cincinnati, OH, USA). MFR was calculated using the following equation:

(1) $$MFR(g/10\min)=\frac{w\times 600}{t}$$

where w is the mass of the melt extruded out of the die of the capillary tube in the MFR tester, g; t stands for the duration to obtain the melt, s.

Five samples were measured and averaged for PBAT and each blend.

#### 2.4.2. Mechanical Characterization

The tensile and flexural tests were performed at room temperature using a universal testing machine (E44.304, MTS Industrial Systems (China) Co., Ltd., Shenzhen, China) with a 20 kN load cell. The tensile test was conducted according to ASTM D 638-22 [14] at a crosshead displacement rate of 50 mm/min, and the three-point flexural test was carried out according to ASTM D 790-2010 [15] at a crosshead displacement rate of 5 mm/min and a span of 80 mm.

A Universal Impact Testing Machine (XJC-25D, Chengde Precision Testing Machine Co., Ltd., Chengde, China) was performed to measure the Charpy impact strength as per Chinese National Standard GB/T 1043.1-2008 [16].

The mean values and standard deviations of all mechanical properties represented an average of five samples.

#### 2.4.3. Scanning Electron Microscopy (SEM) Observation

The morphology of the fractured surface of the specimens during the tensile test was observed using a field-emission scanning electron microscope (SEM, JEOL JSM-7600F, JEOL Ltd., Tokyo, Japan). Before observation, the specimens were sputtered with a thin layer of gold, with current intensity set to 6.8 mA and deposition time to 20 s in vacuum. Images of the cross section was taken at magnification of 1000×. The acceleration voltage of the electrons was set at 3 kV.

#### 2.4.4. Thermal Stability Assessment

The thermal stability of neat PBAT and CS/PBAT blends was analyzed using a thermal gravimetric analyzer (TG209F1, NETZSCH Gerätebau GmbH, Selb, Germany) under nitrogen atmosphere with a flow rate of 20 mL/min. The experiments were performed on 3–5 mg sample from 30 °C to 800 °C with a heating rate of 20 °C/min. Thermogravimetric (TG) curves and derivative thermogravimetric (DTG) curves were recorded and analyzed using NETZSCH software. The 5% weigh loss temperature (T_i_) collected from the TG data, and the maximum degradation temperature (T_p_) collected from the derivative DTG data were used to distinguish differences arising from CS dosage.

#### 2.4.5. Melting and Crystallization Behavior Analysis

The melting and crystallization behavior of neat PBAT and CS/PBAT blends were determined on 3–5 mg samples using a differential scanning calorimeter (DSC), model NETZSCH DSC214 (NETZSCH-Gerätebau GmbH, Selb, Germany). The experiment was performed from 20 °C to 220 °C with a heating rate of 10 K/min and holding at 220 °C for 5 min. Subsequently, the samples were cooled to room temperature with a rate of 10 K/min, before finally being heated again to 220 °C. DSC analysis was performed under nitrogen atmosphere with flow rate of 20 mL/min.

The melt temperature (T_m_) and cold crystallization temperature (T_cc_) were determined based on the DSC test. The crystallinity value (χ_c_) was calculated using the following equation [17,18,19]:

(2) $$x_{c}=\frac{\left|\Delta H_{m}+\Delta H_{cc}\right|}{\omega \Delta H^{\theta }}\times 100\%$$

where $\Delta H^{\theta }$ is the standard molar melting enthalpy referred to a hypothetical 100% crystalline sample. According to the literature, $\Delta H^{\theta }$ of 100% crystalline PBAT is estimated to be 114 J/g [20]. $\Delta H_{m}$ and $\Delta H_{cc}$ are, respectively, the molar enthalpy and the cold crystallization enthalpy experimentally detected on the sample under examination. ω denotes the weight fraction of the polymer under examination with respect to the total weight of the blend.

#### 2.4.6. Water Contact Angle (WCA) Inspection

The wetting behavior and hydrophilicity of the sample surfaces were evaluated by static water contact angle (WCA) measurements using the sessile drop method.

Measurement was carried out using a contact angle instrument (DSA100; KRÜSS GmbH, Borsteler Chaussee, Hamburg, Germany). The contact angle (θ) was measured by dropping 5 µL droplet of distilled water onto the surface and kept for 15 s, followed by reading the θ values at least five different locations.

#### 2.4.7. Mass Change Examination

Weight measurement was performed using an analytical balance before and after degradation tests in water and open-air storage. The mass change of each sample was calculated using the formula below:

(3) $$mc(\%)=\frac{w_{t}-w_{0}}{w_{0}}\times 100\%$$

where mc stands for the weight change during the test,%; $w_{t}$ is the weight of the sample after degradation in water or air for 60 days, g; and $w_{0}$ is the weight of the sample before degradation, g.

## 3. Results and Discussion

### 3.1. Effect of CS Dosage on Properties of the CS/PBAT Blends

#### 3.1.1. Flow Abilities

**Figure 2:**
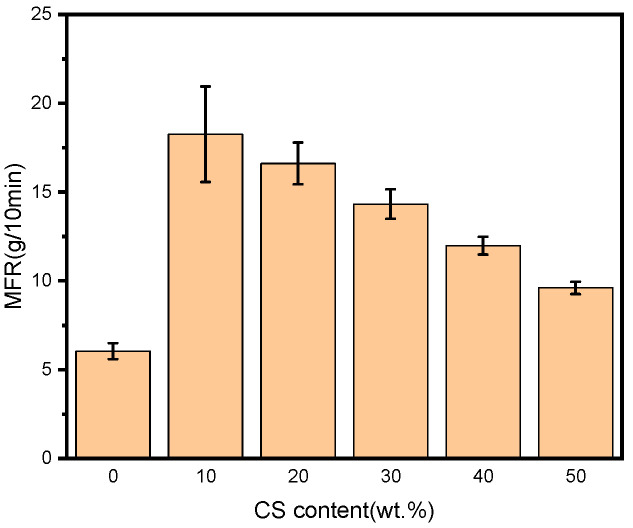
Melt flow rates of neat PBAT and CS/PBAT blends.

The melt flow rates of the blends with different CS contents are demonstrated in Figure 2. From the figure, neat PBAT had a small MFR of 6.04 g/10 min, sharing the same order of magnitude with the result reported by Muthuraj et al. [21]. This might be attributed to the higher degree of polymerization and a relatively narrow molecular weight distribution of PBAT, leading to a weaker movement ability of chain segments. After blending with starch, MFR became greater. As reported by Nomadolo et al. [22], this might be due to the presence of starch in the PBAT matrix, which degrades at higher temperatures. In addition, the blend containing 10 wt.% CS had the greatest MFR of 18.25 g/10 min, increasing by 202.15% compared to that of neat PBAT. The reason should be that a small amount of starch could play the role of separating the PBAT molecular chains, reducing the inter-molecular actions and improving the movement abilities of the chain segments. When too much starch was used, however, some amount of starch might be detained between the polymer melt and the internal wall of the capillary tube during extrusion, which would prevent the flow of the melt and make MFR decrease. The MFR of the 50%CS/PBAT blend reduced to 9.60 g/10 min, which was much smaller than that of 10%CS/PBAT but was still greater than that of BPAT by 58.94%, meaning that the 50%CS/PBAT blend could flow more smoothly than virgin polymer during processing.

#### 3.1.2. Mechanical Properties

**Figure 3:**
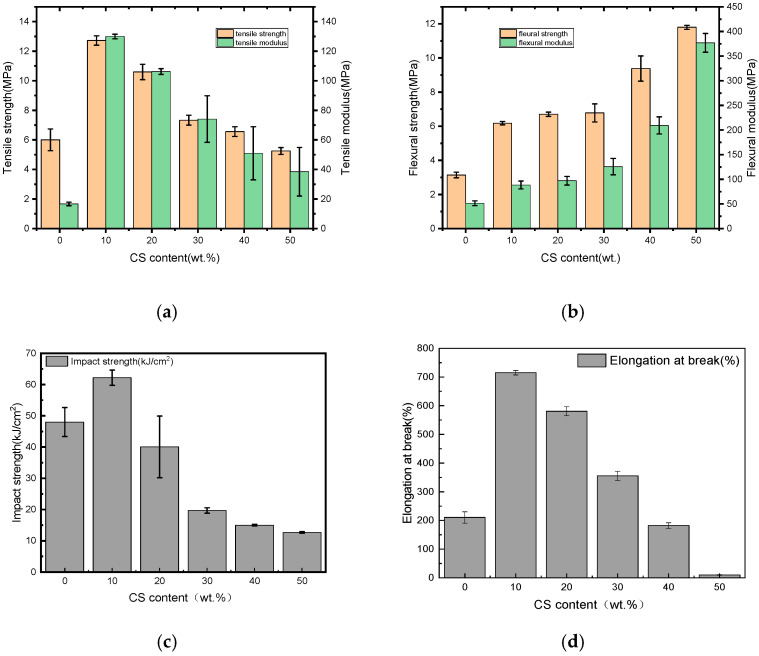
Mechanical properties of neat PBAT and CS/PBAT blends: (**a**) tensile strength and modulus; (**b**) flexural strength and modulus; (**c**) impact strength; (**d**) elongation at break.

**Figure 4:**
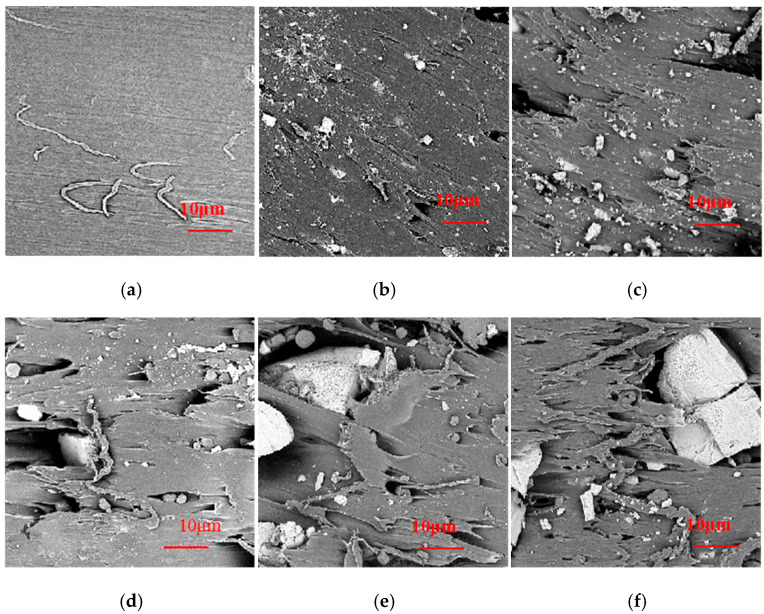
SEM images showing the cross-sectional surfaces of the samples (1000×): (**a**) PBAT; (**b**) 10%CS/PBAT; (**c**) 20%CS/PBAT; (**d**) 30%CS/PBAT; (**e**) 40%CS/PBAT; (**f**) 50%CS/PBAT.

The results of the mechanical properties of the experimental materials were shown in Figure 3. It indicated that the blend containing 10 wt.% starch had a superior tensile strength of 12.72 MPa and tensile modulus of 128.04 MPa compared to the base material (PBAT), which could potentially have a significant reinforcing effect. But more substitution of PBAT with starch would weaken the tensile properties gradually. As reported, the mechanical properties of a multiphase polymer blend was strongly affected by the morphology of the dispersed phase [23]. The cross-sectional fracture surfaces of the tensile samples observed by SEM are illustrated in Figure 4. From Figure 4a, the fracture surface of neat PBAT was smooth. When a small amount of CS was blended with PBAT, the starch was sheared into smaller particles, acted as the dispersed phase, and well distributed in and tightly wrapped with PBAT, as shown in Figure 4b. Besides these, there always existed much hydrogen bonds in the molecular chains of the starch, which made starch strong enough to reinforce PBAT. Consequently, the sample became more resistant to the tension damage, leading to improved tensile properties [24]. When more starch was applied, however, the size of the starch grew because of aggregation. The big-sized starch could not be distributed homogeneously in the matrix, leading to disrupted uniformity of the blend [23] and imperfect interface adhesion between the two polymers. A built-in stress was thus produced and the blends were easier to be fractured during the mechanical test. The defects that exist in the blends containing more starch can be evidenced in the SEM images shown in Figure 4c–f. Some starch debonded from PBAT in the cross-sectional surface of the 20%CS/PBAT blend; meanwhile, the surface became a little coarse but was generally homogeneous. With the percentage of starch in the blends increasing from 20% to 50%, starch appeared in greater sizes, and the fracture surfaces became rougher. In addition, defects became more distinct.

The change trends of the flexural properties differed from those of the tensile ones, as shown in Figure 3b. Both the flexural strength and flexural modulus of the blends always increased with the dosage of starch. The flexural strength and flexural modulus of the blend containing 50% starch were as high as 11.81 MPa and 373.28 MPa, remarkedly increasing from those of PBAT by 285.95% and 646.26%, respectively. This difference might arise from the different fracture mechanisms between the tensile and the flexural tests. The tensile samples failed mainly because of the tensile force in the middle, while the load in a three-point bending test was the combination of the compressive force at the top, the shear force in the middle, and the tensile force at the bottom [25]. The upper and lower surfaces of the specimen under three-point bending were subjected to shear stress [26].

Figure 3c illustrates the effects of the starch dosage on the impact strengths of the blends. Neat PBAT had an impact strength as high as 48.00 kJ/m^2^, meaning that it was highly flexible; this performance has already been reported by Ding et al. [5]. For 10%CS/PBAT, the impact strength rose by 40.02% to 67.21 kJ/m^2^; this significant increase in the impact strength was evidence that the introduction of a small amount of starch could cause PBAT to become tougher. As discussed before, a small amount of starch could be distributed homogeneously in the matrix and acted as a lubricant agent in the surrounding PBAT resin. More energy would be absorbed when the sample was exerted with a force; consequently, the blend showed more flexibility in this situation. When more starch was blended, they would aggregate to form big-sized particles, leading to poor interfacial compatibility between starch and PBAT, after which phase separation occurred. As a result, the impact strength became lower, with the blend containing 50 wt.%CS exhibiting much lower impact strength than its parent polymer; but it was still as high as 12.70 kJ/m^2^, which ensured its safe application in many areas.

The effects of the starch dosage on the elongation at break (EAB) of the blends is demonstrated in Figure 3d, with EAB initially increasing before decreasing. 10%CS/PBAT had an EAB of 715.05%, increasing from that of PBAT by 239.97%, whereas 50%CS/PBAT dropped significantly to 9.95%. This great reduction in EAB was attributed to the much stiffer form of starch than that of PBAT. As a biodegradable polyester, PBAT has been widely used to produce agriculture film because of its excellent ductility. The increased EAB of 10%CS/PBAT meant that the incorporation of a small amount of starch can not only reduce the cost of the film, but it also retain the good ductility of the film.

#### 3.1.3. Thermal Stability

Thermogravimetric analysis (TGA) measures the amount and rate of change in the weight of a material as a function of temperature or time in a controlled atmosphere. It can be used to assess the composition of materials and to predict their thermal stability [26].

**Figure 5:**
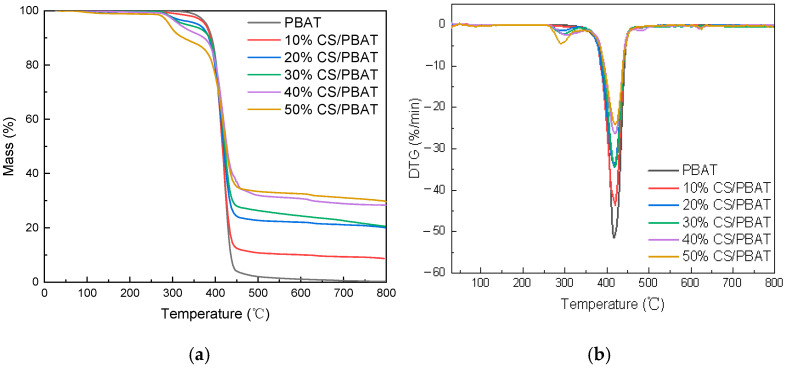
Thermogravimetric analysis of neat PBAT and CS/PBAT blends under nitrogen atmosphere: (**a**) TG curves of composites; (**b**) DTG curves of composites.

The TGA and corresponding derivative thermogravimetric (DTG) curves of neat PBAT and its blends with starch are shown in Figure 5, illustrating the impact of starch content on the thermal stability of the blends. It was found that there was a slight mass loss within 100 °C for all the samples; this loss was associated with the vaporization of free/bound water and PBAT decomposition [2,27]. The mass losses mainly occurred between 200 and 500 °C. For a better understanding on the thermal stabilities of PBAT and CS/PBAT blends, the technical information from Figure 5 is summarized in Table 1.

From Figure 5, PBAT was found to degrade by only one step. Its thermal decomposition would occur at 369.38 °C (T_i_) and end at 450.02 °C (T_f_); the fastest decomposition took place at 420.41 °C (T_p,2_). This single-step decomposition was also observed by Giri et al. [28]; in their research, the decomposition of PBAT was reported to start at around 333 °C, and the maximum degradation occurred at 424 °C.

The 10%CS/PBAT blend showed a similar degradation mechanism as that of pure PBAT, where only one step decomposition could be observed. When more starch was adopted, however, a new peak at around 293 °C appeared in the DTG curve beside the one near 420 °C, corresponding to the decomposition of PBAT. This new peak was attributed to the decomposition of starch in the blend. This distinct separation between the two peaks meant that there existed immiscible phases in the blends when 20 wt.% or more CS was contained.

From Figure 5 and Table 1, it could also be found that all the blends decomposed at lower initial temperatures when compared with the pure PBAT matrix, which indicated worse thermal stability. The T_i_ values of 10%CS/PLA, 20%CS/PLA, 30%CS/PLA, 40%CS/PLA and 50%CS/PLA were 362.50 °C, 362.23 °C, 360.01 °C, 359.34 °C, and 357.39 °C, respectively, which was a variance of 4–12 °C from the T_i_ value of neat PBAT. In addition, the gradually decreased T_i_ values indicated that a greater dosage of CS would lead to a higher thermal unstability of the blend. This was due to the fact that CS was more thermally unstable than PBAT. The poorer thermal stability might be helpful in the degradation of a material under high temperature. In other words, the CS/PBAT blend should be easier to degrade with heat than PBAT.

#### 3.1.4. Melting and Crystallization Behavior

**Figure 6:**
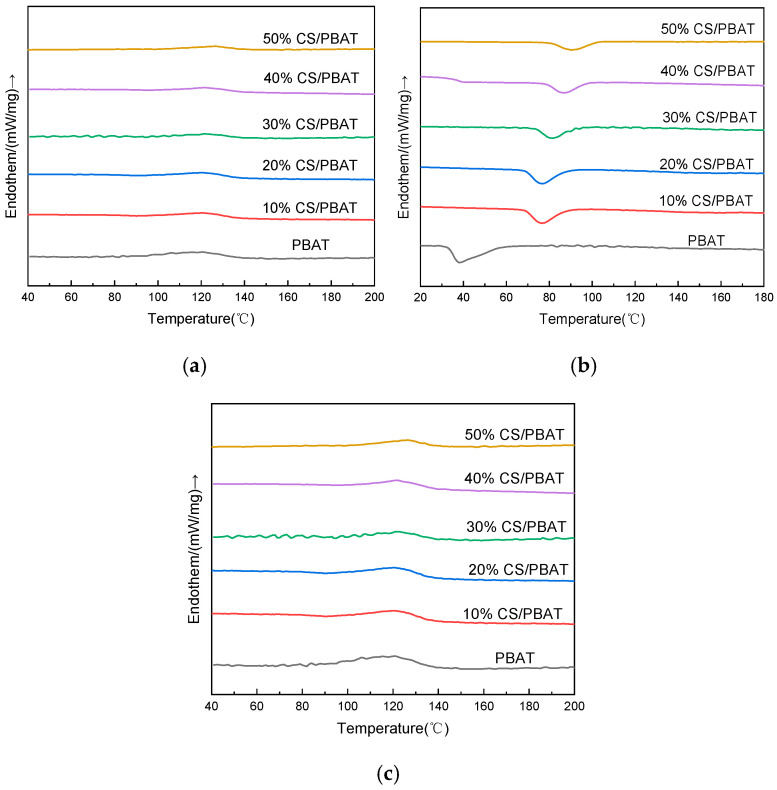
DSC thermograms of neat PBAT and CS/PBAT blends: (**a**) first heating up; (**b**) cooling; (**c**) second heating up.

The DSC thermograms of neat PBAT and CS/PBAT blends are presented in Figure 6. According to the DSC results, the thermal transitions and crystal melting of the samples were assessed, and the derived thermal properties are summarized in Table 2. From Figure 6b, only one peak existed in the cooling curve of each sample, but the peak moved to a higher temperature with the increasing dosage of starch, implying that the incorporation of starch could act as a heterogeneous nucleating agent to promote the crystallization of PBAT and make it easier to crystalize, but this did not change the crystallization mechanism.

The melting temperature (T_m_) of PBAT was 122.7 °C, and it did not experience a notable change with the dosage of starch in the blend, as evidenced in Figure 6c and Table 3. The T_m_ value agreed with that reported in the literature [28,29]. Although the T_m_ values remained constant for PBAT and its blends, the cold crystallization temperature (T_cc_) was found to increase with the CS content, for instance, from 71.3 °C (for pure PBAT) to 90.5 °C (for 50%CS/PBAT). The increase in the T_cc_ value implied that there was an increase in the crystal size; in other words, CS facilitated the crystal growth in the blend [28].

Differing from Figure 6a, Figure 6c shows the second heating curve in which the heat history had been eliminated. From the figure, it could be found that the enthalpy change upon melting (ΔH_m_) gradually became smaller, which was consistent with the results of the crystallinity. When more starch was used, phase separation became more serious, and two continuous phases might be formed. The inter-molecular ordering of PBAT became difficult because of the prevention by the starch continuous phase. Meanwhile, the crystallizable area would be reduced when more PBAT was replaced with starch, leading to a drop in crystallinity, as illustrated in Table 2.

#### 3.1.5. Hydrophilicity

**Figure 7:**
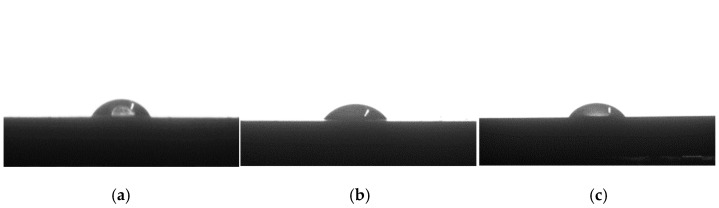

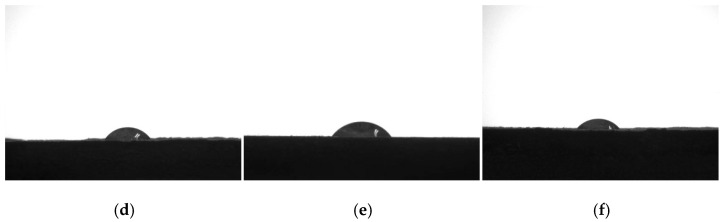
Surface contact angle morphology of the samples: (**a**) PBAT; (**b**) 10%CS/PBAT; (**c**) 20%CS/PBAT; (**d**) 30%CS/PBAT; (**e**) 40%CS/PBAT; (**f**) 50%CS/PBAT.

The hydrophilicity of the samples was assessed by testing its water contact angle (WCA). The effects of starch dosage on the WCAs of the samples are displayed in Figure 7, and the corresponding data are organized in Table 3. PBAT showed a contact angle of 59.9°, while incorporating starch led to the reduction in WCA values, suggesting decreased surface hydrophobicity. In addition, a greater dosage of starch decreased the contact angle to a greater extent. The WCA of 50%CS/PBAT reduced to 32.9° by a marvelous decrease of 45.08% over that of the PBAT. This might have resulted from the inherently high hydrophilic property of starch, which is rich in hydroxyl groups [30]. These hydroxyl groups in the starch structure readily interacted with water molecules, forming hydrogen bonds that enhanced the capacity to absorb the moisture of the sample and increase its overall wettability [3]. Choo et al. [31] also reported the remarkable decrease in the contact angle after the incorporation of starch into PBAT, and the decreased contact angle value implied improved biodegradation behavior of the starch/PBAT mixture with the increased starch hydrolysis rate and hydrophilicity [32]. This enhanced degradation of CS/PBAT blend compared to pure PBAT is also evidenced in our research, as shown in Section 3.3.

### 3.2. Comparative Investigations on Degradation Performances of PBAT and 50%CS/PBAT in Water and Air

As a natural polymer, starch is capable of rapid degradation, owing to its easy utilization by most microorganisms [6,33]. Therefore, filling PBAT with CS should be able to accelerate it degradation. To further understand the degradability of the blend under different conditions, 50%CS/PBAT was chosen as an example because it had the most content of CS among all the samples, and its degradation behaviors in water and in an open-air storage were investigated and compared with those of neat PBAT.

#### 3.2.1. Visual Appearances

**Figure 8:**
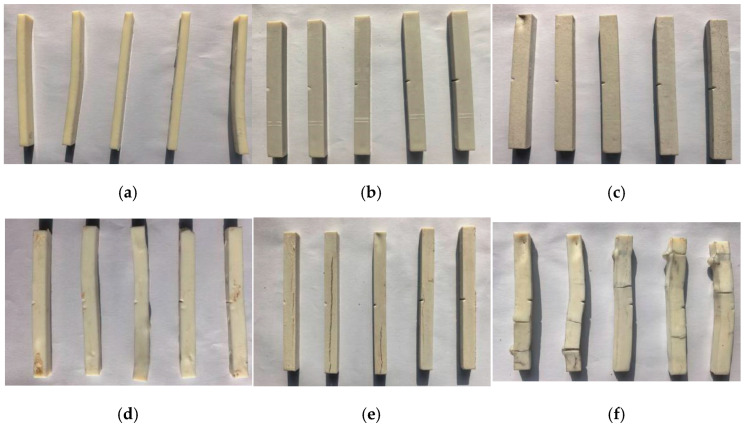
Photographs of neat PBAT and 50%CS/PBAT: (**a**) virgin PBAT, (**b**) PBAT after immersion in water for 60 days, (**c**) PBAT after being placed outdoors in air for 60 days, (**d**) virgin 50%CS/PBAT, (**e**) 50%CS/PBAT after immersion in water for 60 days, (**f**) 50%CS/PBAT after being placed outdoors in air for 60 days.

Figure 8 shows the visual appearances of PBAT and 50%CS/PBAT before the experiment and those in water or air for 60 days. It was found that the appearance of pure PBAT was originally light yellow; immersion in water would blacken the sample, but no any other defects could be observed. For 50%CS/PBAT, its original surface was milky white, owing to the incorporation of white-colored starch into PBAT. After immersion in water for 60 days, the surface color became a bit dim. It was worth noting tha, cracks frequently appeared in the sample. When observing the visual appearances of the samples placed in air, 50%CS/PBAT was also damaged more heavily than virgin PBAT, indicating that the surrounding conditions, such as moisture and sunlight, could degrade 50%CS/PBAT much faster than with PBAT.

#### 3.2.2. Shore Hardness

Hardness of a material is closely related to its intra-structure, its value is affected by various factors, and it is the comprehensive behavior of the microstructure of a material. Table 4 tabulates the measured average Shore hardnesses of the samples under different conditions.

From the table, it could be observed that no obvious differences in Shore hardness occurred between PBAT and 50%CS/PBAT before degradation. After immersion in water for 60 days, however, the Shore hardness of PBAT increased slightly while that of the blend dropped significantly by 10.23%, indicating that water immersion would alter the internal structure of 50%CS/PBAT much heavier than PBAT. In other words, 50%CS/PBAT was easier to degrade in water than neat PBAT; this was consistent with the conclusion from the visual appearance observation. When the samples were placed in air for 60 days, as observed from the table, there exists a very tiny difference in the hardness of PBAT and 50%CS/PBAT, just like that in the original samples, meaning that open-air storage had no obvious effects on the degradation behaviors of the two samples when only the hardness was concerned. The different conclusions from visual appearance observation and the Shore hardness test in air reflected the complexity of the degradation mechanism of a material.

#### 3.2.3. Water Absorption

**Figure 9:**
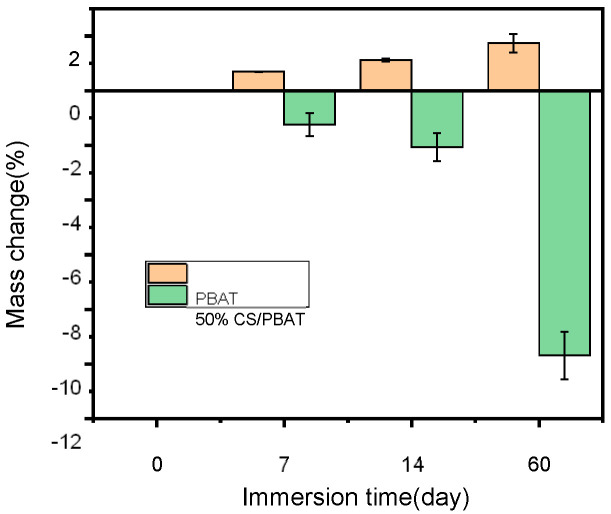
Water absorption of neat PBAT and 50%CS/PBAT.

Water uptakes by PBAT and 50%CS/PBAT at different immersion stages are shown in Figure 9. Generally, PBAT absorbed little water when immersed in water. With the prolonging of the immersion time, its water uptake would increase slightly, up to 1.74%, even after immersion in water for 60 days.

Contrary to that for PBAT, when 50%CS/PBAT was soaked in water, its mass would be lost, and this mass loss became more serious when immersion time extended. After 60 days’ immersion, the mass was reduced by 9.68%. This contrasting mass changing trend with immersion time for PBAT and 50%CS/PBAT might indicate two different action mechanisms of water on the samples. As a plastic, PBAT had a stable molecular structure and was insoluble in water. When it was immersed in water, the molecular structure could not be destroyed but some water molecules might enter the “free volume” among the molecular chains. Thus, the plastic would absorb some water, leading to a positive mass change. For 50%CS/PBAT, however, fifty percent of PBAT was substituted with starch, which was water-soluble. When the blend was immersed in water, some starch would be dissolved in water and the mass of the sample were thus reduced gradually, showing a negative mass change.

### 3.3. Cost Analysis

As mentioned in Section 3.1, the blends, especially 50%CS/PBAT, became much more thermally unstable and hydrophilic. Meanwhile, its degree of crystallinity showed obvious reduction. All these factors were theoretically helpful for its degradation behavior. The investigations in Section 3.2 made evident the fact that 50%CS/PBAT was easier to degrade than neat PBAT, whether in water or in air. All these showed that 50%CS/PBAT should be an ideal degradable material that could be applied in many occasions. Importantly, the high production cost of PBAT is a significant factor limiting its widespread application [34], whereas 50%CS/PBAT has a much lower material cost than pure PBAT.

The current market prices of PBAT and CS in China are about USD 150/ton and USD 30/ton, respectively. The material cost of 50%CS/PBAT would thus be USD 90/ton, representing a reduction of about 40% in costs. This reduced material cost may make the blend more competitive on the market.

Compared with traditional single-component materials for packaging, agricultural mulch, and other applications, the poor interfacial compatibilities in the CS/PBAT blend may worsen some mechanical properties. However, the blend has much better comprehensive properties, such as easiness to degrade and low cost.

## 4. Conclusions

This study successfully produced CS/PBAT blends using a combination of extrusion and injection molding and explored the degradability of the blend in air and in water. The dosage of starch used significantly impacted the comprehensive properties of the blends. The flow ability, tensile strength, tensile modulus, elongation at break, and impact strength all initially increased before decreasing, while the flexural strength and flexural modulus increased monotonously. Notably, increased dosage of CS made the blend become more thermally unstable, hydrophilic, and have a smaller degree of crystallinity, which were all beneficial for the degradation of the blend. 50%CS/PBAT had a much lower material cost and degraded more heavily than neat PBAT, whether in water or in an open-air storage, when their mass change and hardness were concerned. This study demonstrates that the blend made with corn starch and PBAT offers a promising approach for developing new materials with reduced cost and enhanced degradability, making them suitable for applications in many areas, such as packaging and agricultural mulch.

Future work concentrate on the forming properties and durabilities of the film made from the blends.

## Figures and Tables

**Table 1:** Thermogravimetric analysis of neat PBAT and CS/PBAT blends.

Sample	T_i_	T_p,1_	T_p,2_	T_f_
PBAT	369.38		420.41	450.02
10%CS/PBAT	365.20		419.80	449.80
20%CS/PBAT	362.23	293.51	418.89	447.76
30%CS/PBAT	360.01	294.96	417.93	446.59
40%CS/PBAT	359.34	294.39	419.45	444.42
50%CS/PBAT	357.39	292.46	419.09	443.01

**Table 2:** Thermal properties of PBAT and CS/PBAT blends as calculated from the normalized.

DSC Data					
Sample	T_cc_/°C	ΔH_c_ (J/g)	T_m_/°C	ΔH_m_ (J/g)	X_c_/%
PBAT	71.3	−17.39	122.7	13.43	11.78
10%CS/PBAT	76.5	−15.43	123.7	12.04	11.73
20%CS/PBAT	76.3	−13.51	124.3	10.51	11.52
30%CS/PBAT	81.1	−12.43	124.7	8.835	11.06
40%CS/PBAT	87.1	−9.45	125.1	6.85	10.01
50%CS/PBAT	90.5	−9.29 1	125.6	5.58	9.79

**Table 3:** Contact angle of water on the surfaces of neat PBAT and CS/PBAT blends.

Sample	PBAT	10%CS/PBAT	20%CS/PBAT	30%CS/PBAT	40%CS/PBAT	50%CS/PBAT
Contact angle/°	59.9 ± 6.0	55.1 ± 3.5	51.0 ± 2.9	44.9 ± 4.0	40.2 ± 3.1	32.9 ± 1.6

**Table 4:** Average Shore hardnesses of PBAT and 50%CS/PBAT.

	PBAT	50%CS/PBAT
Virgin	42.92 ± 3.36	42.44 ± 2.15
Immersion in water for 60 days	43.60 ± 2.27	38.10 ± 5.84
Placed in air for 60 days	40.18 ± 3.83	40.84 ± 3.93

## Data Availability

The original contributions presented in this study are included in the article. Further inquiries can be directed to the corresponding author.
